# Cardiac Implications of Itraconazole Therapy in Histoplasmosis Patients

**DOI:** 10.7759/cureus.59076

**Published:** 2024-04-26

**Authors:** Rand Sabanci, Moiz Saeed, Kevin Watat, Matthew Wilcox

**Affiliations:** 1 Internal Medicine, Michigan State University, East Lansing, USA; 2 Internal Medicine, Michigan State University College of Human Medicine, East Lansing, USA; 3 Cardiology, Sparrow Hospital Thoracic and Cardiovascular Institute, Lansing, USA

**Keywords:** antifungal drugs, adverse event, itraconazole, heart failure with preserved ejection fraction, pulmonary histoplasmosis

## Abstract

A male patient in his 60s, with a history of tobacco use, presented with fever, weight loss, and cough, and was ultimately diagnosed with histoplasmosis. Initial treatment with itraconazole (ITZ) led to symptom improvement. However, two months later, he returned with lower extremity swelling and dyspnea. Imaging showed pleural effusions and reduced ejection fraction, suggesting itraconazole-induced cardiac toxicity. Transition to voriconazole and initiation of guideline-directed medical therapy improved symptoms. This case report delves into the cardiac side effects of itraconazole, notably heart failure, and elucidates the potential underlying mechanisms. Our goal is to emphasize the importance of monitoring patients on itraconazole for potential cardiac complications, necessitating timely intervention to mitigate adverse outcomes.

## Introduction

Itraconazole (ITZ), a widely employed triazole antifungal for onychomycosis and severe fungal infections, has been associated with various cardiac side effects such as hypertension, cardiomyopathy, reduced ejection fraction, and edema. Despite the incomplete understanding of these effects, multiple theories have been proposed as a potential mechanism. This abstract highlights a case of heart failure with mid-range reduced ejection fraction in a previously healthy individual following the initiation of antifungal therapy with itraconazole.

## Case presentation

A male patient in his 60s, with a past medical history significant for tobacco use disorder and hypertension, presented to the emergency department with complaints of fever, 20 lbs weight loss, and dry, non-productive cough for two weeks. Vitals were significant for fever at 101 F and hypoxia requiring noninvasive mechanical ventilation. His lab workup revealed a sodium level of 127 meq/L, a white blood cell count of 6.6 x103/uL, and a C-reactive protein of 8.1 mg/dL. Computed tomography of the chest showed bilateral nodular lesions and multiple enlarged mediastinal and bilateral hilar lymph nodes (Video 1).

**Video 1:** Computed tomography axial section of the chest showing bilateral nodular lesions and multiple enlarged mediastinal and bilateral hilar lymph nodes

Brain imaging revealed no acute process, and the patient was started on antibiotics with ceftriaxone and azithromycin. Further investigation via bronchoscopy unveiled bilateral lung nodules and lymphadenopathy. Endobronchial ultrasound-guided transbronchial needle aspiration (EBUS-TBNA) of multiple lymph nodes and lower lobe tissue was conducted, with pathology results indicating chronic inflammation and non-necrotizing granulomas and ruling out malignancy. Subsequent respiratory cultures grew Streptococcus mitis and Histoplasma capsulatum. Further extensive fungal workup, including immunodiffusion and complement fixation, revealed reactive titer for histoplasmosis at 1:16 and positive M precipitin bands. An echocardiogram at the time showed a normal ejection fraction at 55-60% with no vegetation seen (Video 2).

**Video 2:** Echocardiogram four-chamber view showing normal ejection fraction at 55-60% with no vegetation seen

The patient was initiated on a six-month course of itraconazole, leading to notable amelioration of clinical symptoms. Upon one-month follow-up, the patient reported complete resolution of symptoms, corroborated by computed tomography findings demonstrating interval improvement in pulmonary nodularity and mediastinal adenopathy (Video 3).

**Video 3:** Computed tomography axial section of the chest showing interval improvement in pulmonary nodularity and mediastinal adenopathy

Two months following the initiation of itraconazole, the patient presented to the emergency department with newly developed lower extremity swelling and exertional dyspnea persisting for two weeks. He was hypoxic on presentation, requiring oxygen supplementation, and his labs were significant for elevated B-type natriuretic peptide at 700 pg/mL, erythrocyte sedimentation rate of 79 mm/L, and white blood cell count of 12.3 x103/uL. A computed tomography scan of his chest revealed worsening bilateral lung nodularity with new left greater than right bilateral pleural effusions (Video 4).

**Video 4:** Computed tomography axial section of the chest showing worsening bilateral lung nodularity with new left greater than right bilateral pleural effusions

**Figure 1:**
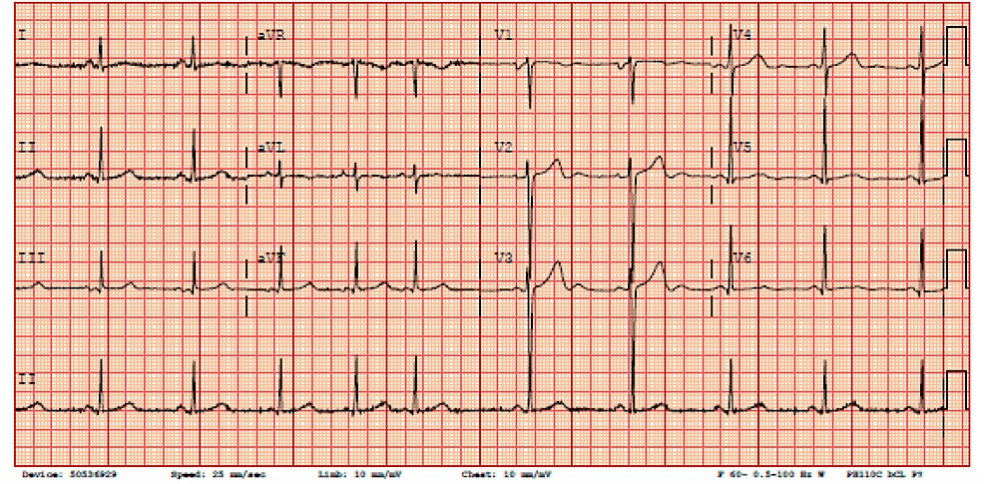
EKG showing sinus rhythm with no ischemic changes

The electrocardiogram showed normal sinus rhythm with no ischemic changes (Figure 1).

A repeat echocardiogram revealed a newly reduced ejection fraction at 40-45% with global hypokinesis and impaired relaxation pattern of left ventricular diastolic dysfunction (Video 5).

**Video 5:** Echocardiogram four-chamber view showing newly reduced ejection fraction at 40-45% with global hypokinesis and impaired relaxation pattern of left ventricular diastolic dysfunction

**Figure 2:**
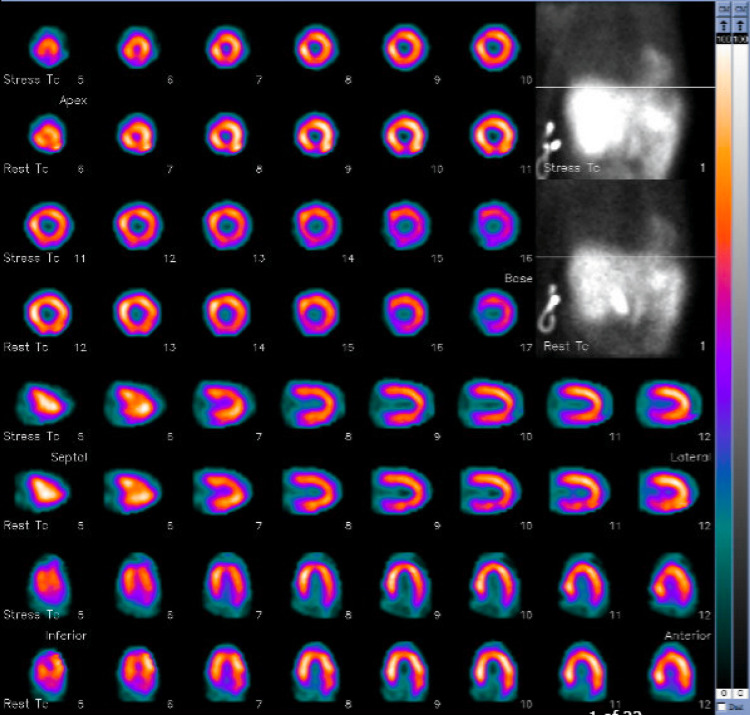
Cardiac stress test with normal findings

The nuclear stress test was normal (Figure 2).

Intravenous diuretic therapy resulted in a significant improvement in the patient's symptoms. Subsequently, the patient was transitioned from itraconazole to voriconazole and commenced guideline-directed medical therapy comprising metoprolol tartrate, sacubitril-valsartan, and dapagliflozin. A three-month follow-up echocardiogram demonstrated improved left ventricular ejection fraction (LVEF) ranging between 55% and 60% and resolution of the left ventricular relaxation pattern.

## Discussion

Itraconazole (ITZ), a triazole antifungal agent, has been a cornerstone in the treatment of various invasive fungal infections, such as aspergillosis, blastomycosis, histoplasmosis, and onychomycosis, since its approval in 1992. Its mechanism of action involves inhibiting the enzyme 14-α- demethylase, thereby impeding the conversion of ergosterol to lanosterol [1].

Despite its efficacy, ITZ is associated with a spectrum of adverse effects, including nausea, vomiting, diarrhea, and transaminitis [2]. Of particular concern are reports of cardiac toxicity linked to ITZ use, leading to a review of the Adverse Event Reporting System (AERS) database in the late 1990s. This review revealed 94 cases of cardiac toxicity from 1992 to 2001, with 58 cases of heart failure possibly associated with ITZ, prompting the issuance of a Food and Drug Administration (FDA) black box warning in 2001 [1].

The precise mechanism underlying ITZ-induced cardiac dysfunction remains elusive. Cleary et al. proposed a likely causal relationship between itraconazole (ITZ) and cardiac dysfunction, supported by ex vivo and in vitro research. They suggested that inhibition of mitochondrial oxidative phosphorylation in myocardial tissue could be a potential mechanism, which seems to be dose-dependent [3]. In contrast, Qu et al. reported a direct negative inotropic effect of ITZ without evidence of mitochondrial dysfunction [4].

A recent comprehensive study conducted in 2020 involving 69 patients shed further light on the cardiac side effects of ITZ. The study found edema to be the most prevalent symptom (74% of patients), with heart failure affecting nearly a quarter of patients, both with and without preserved ejection fraction (19.4% and 22.6%, respectively). Other observed adverse effects included worsening or new hypertension (25.8%), pulmonary edema, pericardial effusion, and one case of cardiac arrest [5]. The median time from initiation of ITZ therapy to the onset of adverse effects was four weeks, with most patients experiencing multiple adverse drug events simultaneously. Clinicians responded by discontinuing (74%) or reducing (19%) ITZ dosage. Resolution of symptoms was achieved in 54% of cases, with partial resolution in 30% [5]. However, in the subset of patients with heart failure with reduced ejection fraction (HFrEF), resolution was less consistent [5].

Given the lack of systematic studies, no precise risk estimate for ITZ-induced heart failure can be provided. Nevertheless, precautionary measures have been recommended, including close monitoring for signs and symptoms of heart failure during ITZ therapy and considering contraindications in patients with evidence of ventricular dysfunction [1]. In such cases, alternative antifungal agents like voriconazole, which lacks reported associations with cardiac adverse effects, may be considered [1]. As further research unravels the intricacies of itraconazole’s side effects, clinicians must remain vigilant, opting for alternative therapies when warranted, and diligently monitoring patients for signs of cardiac compromise to ensure optimal therapeutic outcomes.

## Conclusions

This case underscores the potential link between the administration of itraconazole and the onset of congestive heart failure (CHF). Establishing a definitive causal relationship is challenging due to the scarcity of scientific data on the matter. Hence, it is imperative to uphold pharmacovigilance and conduct observational studies to thoroughly evaluate this association. Nevertheless, given the current evidence, it is prudent to incorporate awareness of this adverse effect into clinical practice and contemplate its contraindication in patients with a history of heart failure or left ventricular dysfunction. In such scenarios, voriconazole presents itself as a viable alternative, offering efficacy against Histoplasma sp. without reported associations with heart failure development.
